# Serum Leucine-Rich *α*2-Glycoprotein as a Biomarker for Monitoring Disease Activity in Patients with Systemic Juvenile Idiopathic Arthritis

**DOI:** 10.1155/2019/3140204

**Published:** 2019-02-04

**Authors:** Masaki Shimizu, Natsumi Inoue, Mao Mizuta, Yasuo Nakagishi, Akihiro Yachie

**Affiliations:** ^1^Department of Pediatrics, School of Medicine, Institute of Medical, Pharmaceutical, and Health Sciences, Kanazawa University, Kanazawa, Japan; ^2^Department of Pediatric Rheumatology, Hyogo Prefectural Kobe Children's Hospital, Kobe, Japan

## Abstract

To investigate whether serum leucine-rich *α*2-glycoprotein (LRG) levels are useful as a marker of disease activity in systemic juvenile idiopathic arthritis (s-JIA), we determined serum LRG levels in fifty-nine s-JIA patients, 15 with other subtypes of JIA, 7 with Kawasaki disease (KD), 7 with influenza A infection (flu), 7 with enterohemorrhagic *Escherichia coli* (EHEC) infection, and 20 healthy controls (HC). Results were compared with the clinical features of s-JIA and serum cytokine levels including interleukin- (IL-) 6, IL-18, and soluble tumor necrosis factor receptors I and II. Serum LRG levels in active s-JIA were higher compared to those in other subtypes of JIA, EHEC, flu patients, and HC. Serum LRG levels were normalized in the inactive s-JIA phase after treatment. Serum LRG levels were positively correlated with serum C-reactive protein and ferritin levels. Serum LRG levels reflected s-JIA disease activity and thus may be useful for monitoring s-JIA disease activity.

## 1. Introduction

Systemic juvenile idiopathic arthritis (s-JIA) is a systemic inflammatory condition characterized by arthritis and other systemic features, including spiking fever, salmon-colored skin rash, hepatosplenomegaly, generalized lymphadenopathy, and serositis [1]. Systemic inflammation in s-JIA is closely associated with the dysregulation of the innate immune system driven by proinflammatory cytokines. In particular, IL-1, IL-6, TNF-*α*, and IL-18 play a major role in the pathogenesis of s-JIA [1].

Leucine-rich *α*2-glycoprotein (LRG) is a plasma glycoprotein containing repetitive sequences with a leucine-rich motif. The physiological role of LRG remains obscure, but recent studies revealed that LRG promotes the differentiation and the proliferation of Th17 [2] and neovascularization through causing a switch in transforming growth factor beta (TGFbeta) signaling in endothelial cells [3]. LRG is mainly produced in the liver and neutrophils [4]. The expression of LRG is increased in various inflammatory conditions [5–8]. Proinflammatory cytokines, including interleukin- (IL-) 1*β*, IL-6, and tumor necrosis factor (TNF)-*α*, can upregulate LRG expression [9]. Recent studies showed that LRG is also one of the clinically useful acute-phase reactants in various inflammatory diseases including inflammatory bowel diseases [5, 6], rheumatoid arthritis [7], and adult-onset Still's disease [8].

In this study, to investigate whether serum LRG levels reflect the disease activity of s-JIA, we measured serum LRG levels in s-JIA patients and determined their correlation with disease activity.

## 2. Materials and Methods

### 2.1. Patients and Samples

Serum samples were obtained from 59 active s-JIA patients (male/female = 29/30, mean age: 6.0 years), 5 patients with rheumatoid factor-positive polyarticular JIA (RF+ polyJIA) (2/3, 12.4), 5 patients with oligoarticular JIA (oligoJIA) (2/3, 5.8), 5 patients with enthesitis-related arthritis (ERA) (3/2, 13), 7 patients with Kawasaki disease (KD) (1/6, 2.9), 7 patients with influenza A infection (flu) (1/6, 4.4), 7 patients with enterohemorrhagic *Escherichia coli* infection (EHEC) (4/3, 8.7), and 20 healthy controls (HC) (12/8, 7.4). Eleven s-JIA patients developed a complication of macrophage activation syndrome (MAS). Eight s-JIA patients were longitudinally evaluated in the active, inactive, and remission phases. Four s-JIA patients were evaluated longitudinally on a second occasion when their disease was in an inactive phase. Therefore, twelve patient data points in the inactive phase and eight patient data points in the remission phase could be evaluated. The clinical characteristics of 59 active s-JIA patients are shown in Table 1. Of a total of 59 patients, 45 were newly diagnosed and not given treatment. Fourteen were the patients with relapse during the treatment with prednisolone (PSL). In addition to PSL, three patients were also treated with cyclosporine and one was treated with methotrexate and tacrolimus. Samples from the patients with relapse were obtained at the time of the diagnosis of relapse.

Diagnoses of s-JIA and other types of JIA were based on the International League of Associations for Rheumatology criteria [10]. MAS was diagnosed based on the 2016 EULAR/ACR/PRINTO classification criteria [11]. The criteria defining the active phase of s-JIA were as follows: fever and a single feature including active arthritis, rash, hepatosplenomegaly, generalized lymphadenopathy, and serositis, along with increased erythrocyte sedimentation rates and CRP levels. Patients with sepsis or severe bacterial infection were excluded. Some patients had minimal joint disease at the onset of s-JIA, and the presence of arthritis was confirmed later. The criteria for the inactive phase of s-JIA in patients on medication were as follows: the first time with no clinical symptoms that were observed in the active phase, as well as normal erythrocyte sedimentation rates (<5 mm/h) and CRP levels (<0.1 mg/dl). The criterion for remission of patients with s-JIA on medication was 6 continuous months of inactive disease while receiving treatment. Diagnosis of KD was based on the classic clinical criteria [12]. The diagnosis of EHEC O111 infection was based on microbiological identification of EHEC. The diagnosis of flu was based on the detection of influenza antigen in nasopharyngeal swabs. Samples from the patients form other types of JIA, KD, EHEC, and flu were obtained at the diagnosis of each disease before treatment.

Serum was separated from cells, divided into aliquots, frozen, and stored at −80°C until use. This study was approved by the Institutional Review Board at Kanazawa University, and all specimens were used after informed consent was obtained.

### 2.2. Measurement of Serum LRG and Cytokine Levels

Serum LRG, IL-6, IL-18, and soluble tumor necrosis factor receptor (sTNFR) I and II levels were measured using commercial enzyme-linked immunosorbent assay according to the manufacturer's instructions (LRG: IBL, Fujioka, Japan; IL-18 and IL-6: MBL, Nagoya, Japan; and soluble TNF-*α* receptor types I and II: R&D Systems Inc., Minneapolis, MN, USA).

### 2.3. Statistical Analysis

Multiple comparisons among groups were analyzed using Tukey's test. The comparison between the active phase and inactive phase, the active phase and remission, and the inactive phase and remission in each patient was analyzed using the paired *t*-test. Correlations were expressed using Spearman's rank correlation coefficient. For the analyzed measures, *p* values less than 0.05 were considered significant.

## 3. Results and Discussion

### 3.1. Serum LRG Levels in Various Inflammatory Diseases

**Figure 1:**
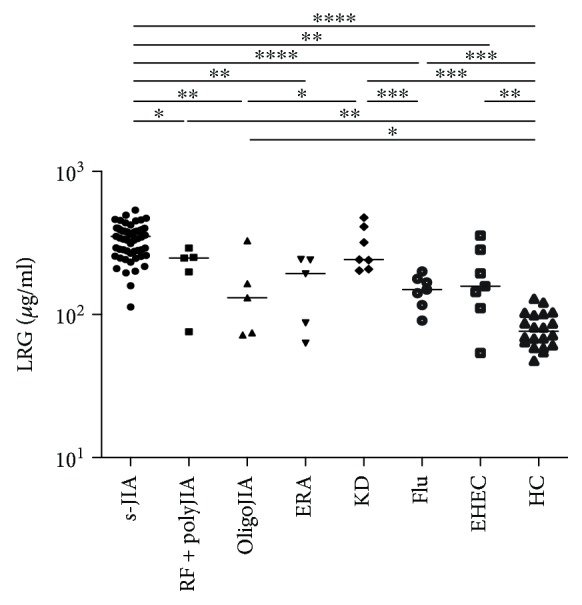
Serum levels of LRG in patients with s-JIA and other various diseases. Bars represent median values. Statistically significant differences between each patient group are shown as ^∗^*p* < 0.05, ^∗∗^*p* < 0.01, ^∗∗∗^*p* < 0.01, and ^∗∗∗∗^*p* < 0.0001. LRG: leucine-rich *α*2-glycoprotein; s-JIA: systemic juvenile idiopathic arthritis; RF+ polyJIA: rheumatoid factor-positive polyarticular JIA; oligoJIA: oligoarticular JIA; ERA: enthesitis-related arthritis; KS: Kawasaki disease; flu: influenza A infection; EHEC: enterohemorrhagic *Escherichia coli* infection; HC: healthy controls.

We measured serum LRG levels in s-JIA patients and compared these with those of patients with other subtypes of JIA, KD, flu, or EHEC. Compared with those in HC (median, 76.2; range, 47.4–128.8 *μ*g/ml), serum LRG levels were significantly elevated in patients with s-JIA (349.5; 113.0-537.0 *μ*g/ml, *p* < 0.0001), RF+ polyJIA (247.5; 75.8–291.0 *μ*g/ml, *p* < 0.01), oligoJIA (131.1; 71.9-328.4 *μ*g/ml, *p* < 0.05), KD (241.8; 203.4-475.2 *μ*g/ml, *p* < 0.001), flu (149.2; 90.8-199.6 *μ*g/ml, *p* < 0.001), and EHEC (157.5; 53.9-355.1 *μ*g/ml, *p* < 0.01), as shown in Figure 1. Serum LRG levels were significantly elevated in s-JIA compared with RF+ polyJIA (*p* < 0.05), oligoJIA (*p* < 0.01), ERA (*p* < 0.01), flu (*p* < 0.0001), and EHEC (*p* < 0.01) and were significantly higher in KD than in oligoJIA (*p* < 0.05) and flu (*p* < 0.001).

### 3.2. Time Course of Changes in Serum LRG Levels in s-JIA Patients

**Figure 2:**
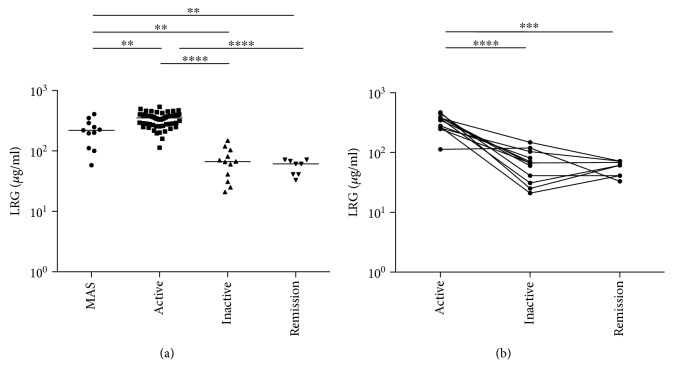
Changes in serum LRG in patients with s-JIA. (a) Serum LRG levels in each s-JIA phase are shown. (b) Changes in serum LRG level are shown. Statistically significant differences between each patient group are shown as ^∗∗^*p* < 0.01, ^∗∗∗^*p* < 0.01, and ^∗∗∗∗^*p* < 0.0001. LRG: leucine-rich *α*2-glycoprotein.

We compared serum LRG levels in each phase of s-JIA. As shown in Figure 2(a), serum LRG levels were significantly elevated during the active phase compared with the MAS (*p* < 0.01), inactive (*p* < 0.0001), and remission phases (*p* < 0.0001). Serum LRG levels were significantly elevated during the MAS phase compared with the inactive (*p* < 0.001) and remission phases (*p* < 0.001). To investigate the relevance of serum LRG levels in the pathogenesis of s-JIA, serum LRG levels were serially monitored in 12 (8 + 4) cases of s-JIA (Figure 2(b)). Serum LRG levels in s-JIA patients decreased to the levels in HC in the inactive phase.

### 3.3. Correlation between Serum LRG Levels and Measures of Disease Activity in s-JIA Patients

**Figure 3:**
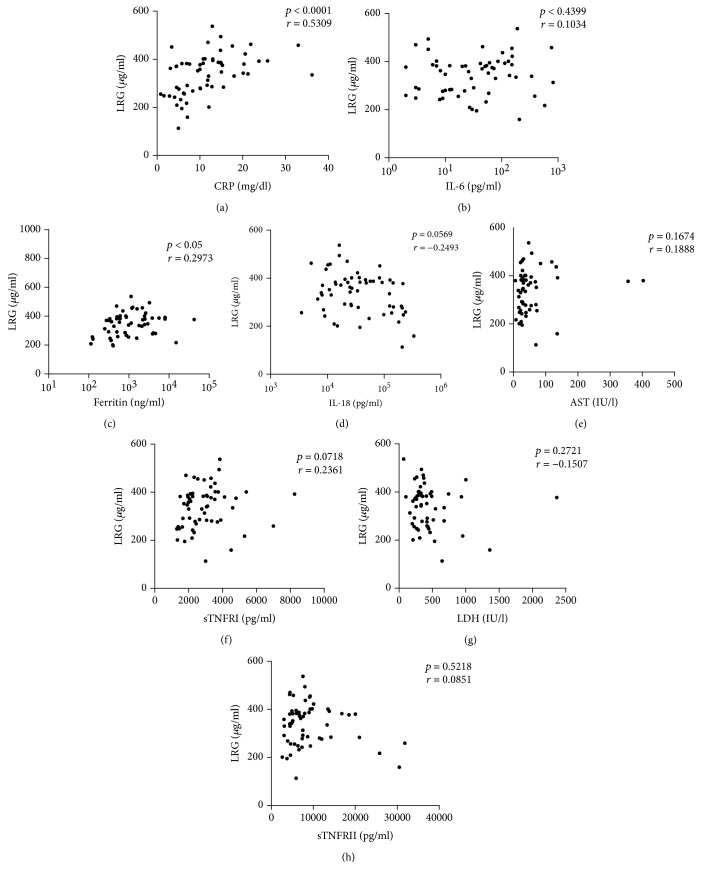
Correlations between serum LRG levels and other measures of disease activity. Serum LRG levels were compared with other serum biomarker levels in s-JIA patients. (a) CRP, (b) ferritin, (c) AST, (d) LDH, (e) IL-6, (f) IL-18, (g) sTNFRI, and (h) sTNFRII. Black boxes indicate the values in s-JIA patients. LRG: leucine-rich *α*2-glycoprotein; CRP: C-reactive protein; AST: aspartate aminotransferase; LDH: lactate dehydrogenase; IL: interleukin; sTNFR: soluble tumor necrosis factor receptor.

We assessed the correlation of serum LRG levels to disease indicators and proinflammatory cytokines. Serum LRG levels correlated positively with serum CRP levels (Figure 3(a)) and ferritins (Figure 3(b)); however, it was not correlated with other disease indicators (Figure 3(c)–3(h)).

## 4. Discussion

LRG is an acute-phase reactant which is induced by proinflammatory cytokines in various inflammatory diseases [5–8, 13]. Previous studies revealed that serum LRG levels are elevated not only in infectious diseases but also in rheumatic diseases such as KD [13], rheumatoid arthritis (RA) [7], and adult-onset Still's disease [8], which is regarded as the adult manifestation of a disease spectrum that includes s-JIA.

In this study, we demonstrated that serum LRG levels were elevated in the active phase of s-JIA and other subtypes of JIA. In s-JIA patients, serum LRG levels were significantly elevated during both the active phase and MAS phases and normalized in the inactive phase. These findings indicate that serum LRG levels are useful for monitoring disease activity in s-JIA.

Serum LRG levels were positively correlated with serum CRP and ferritin levels but had no correlation with other indicators of s-JIA disease activity. The reason for the absence of correlation between serum LRG levels and the indicators of s-JIA disease activity other than CRP and ferritin is unclear. However, some s-JIA patients in this study were treated with steroid and/or immunosuppressants. Our previous study showed that the kinetics of serum LRG levels after starting anti-inflammatory treatment was different from that of other indicators [14]. Therefore, these treatments may also have some effect causing this discrepancy. Serum LRG levels were significantly elevated in the active phase of s-JIA compared to those in the MAS phase. It might be because most of the patients with MAS were on medication including steroids and/or cyclosporine.

The induction mechanisms of LRG are different from those of CRP. CRP expression is essentially dependent on IL-6. On the other hand, LRG expression is also induced by IL-6 and upregulated synergistically with either IL-1*β* or TNF-*α* [15]. Furthermore, IL-1*β*, TNF-*α*, and IL-22 can induce LRG expression without the presence of IL-6 [6]. These findings indicate that LRG is different from CRP and is a unique acute reactant.

Tocilizumab (TCZ), a humanized anti-IL-6 receptor monoclonal antibody, has a dramatic effect on s-JIA patients [15]. However, CRP measurement is not useful during TCZ therapy because IL-6 is the chief stimulator of CRP production [16]. Therefore, a new biomarker to identify patients whose disease remains active even with IL-6 inhibition is needed. We previously reported 4 s-JIA patients whose serum LRG level is useful as a marker of disease activity during IL-6 blockade treatment [14]. A further large study with a greater number of s-JIA patients receiving TCZ is necessary to confirm the clinical usefulness of serum LRG levels as a biomarker for evaluating s-JIA disease activity during TCZ therapy.

In conclusion, serum LRG levels reflected s-JIA disease activity. Monitoring of serum LRG levels may be useful for assessing s-JIA disease activity, although further large studies with a greater number of s-JIA patients including those receiving TCZ therapy are necessary to confirm the clinical usefulness of serum LRG levels as a biomarker for evaluating s-JIA disease activity.

## Figures and Tables

**Table 1:** Clinical characteristics of patients with systemic juvenile idiopathic arthritis during the active phase.

	s-JIA
Patients	59
Sex (male/female)	29/30
Age (years)	6.0 (0.7-26)
Disease duration (months)	2.0 (0-82)
Clinical symptoms	
Fever (%)	59 (100)
Rash (%)	44 (74.6)
Hepatomegaly (%)	7 (11.9)
Splenomegaly (%)	3 (5.1)
Lymphadenopathy (%)	13 (22.0)
Serositis (%)	5 (9.5)
Arthritis (%)	32 (54)
Laboratory findings	
CRP (mg/dl) (normal < 0.3)	10.8 (0.85-36.2)
AST (IU/l) (normal 13-33)	31 (6-403)
LDH (IU/l) (normal 119-229)	344 (70-1359)
Ferritin (ng/ml) (normal 10-280)	977 (113-42490)
Treatments	
Prednisolone (mg/kg/day) (*n* = 14)	0.45 (0.12-2.0)
Cyclosporine A (mg/kg/day) (*n* = 3)	4 (4-5)
Methotrexate (mg/week) (*n* = 1)	5
Tacrolimus (mg/day) (*n* = 1)	1.5

CRP: C-reactive protein; AST: aspartate aminotransferase; LDH: lactate dehydrogenase.

## Data Availability

All data generated or analyzed during this study are included in this published article.

## Abbreviations

LRG: Leucine-rich *α*2-glycoprotein
s-JIA: Systemic juvenile idiopathic arthritis
KD: Kawasaki disease
flu: Influenza
EHEC: Enterohemorrhagic *Escherichia coli*
HC: Healthy control
IL: Interleukin
TNF: Tumor necrosis factor
ERA: Enthesitis-related arthritis
MAS: Macrophage activation syndrome
PSL: Prednisolone.
